# Effects of the umbilical cord mesenchymal stem cells in the treatment of knee osteoarthritis: A systematic review and meta-analysis

**DOI:** 10.1097/MD.0000000000040490

**Published:** 2024-11-15

**Authors:** Zhijian Xiao, Xinying Wang, Cheng Li, Lihua Luo, Wei Li

**Affiliations:** a Hangzhou Fuyang Hospital of TCM Orthopedics and Traumtology, Hangzhou, China; b Hangzhou Tongjuntang Second TCM Clinic, Hangzhou, China; c Hangzhou Tongyuderen TCM Clinic, Hangzhou, China; d The Second School of Clinical Medicine, Zhejiang Chinese Medical University, Hangzhou, China.

**Keywords:** knee osteoarthritis, meta-analysis, systematic review, umbilical cord mesenchymal stem cells

## Abstract

**Background:**

This study aimed to evaluate the effects of umbilical cord mesenchymal stem cells (UC-MSCs) in the treatment of knee osteoarthritis.

**Methods:**

PubMed, Web of Science, Cochrane Library, Embase, Chinese National Knowledge Infrastructure and Wanfang databases were searched from inception to March 31, 2024. *RevMan 5.3* was used to conduct meta-analyses of the final included studies.

**Results:**

Three randomized controlled studies were conducted. Western Ontario and McMaster Universities Osteoarthritis Index was reduced in the UC-MSCs group compared that in to the control group (mean difference: ‐25.85; 95% confidence interval: −41.50, −10.20; *P* = .001). Knee Lysholm Score was improved in the UC-MSCs group compared with the control group (mean difference: 18.33; 95% confidence interval: 12.89, 23.77; *P* < .00001). Egger test showed *P* = .583, indicating no publication bias. Sensitivity analysis indicated that the results were stable.

**Conclusion:**

Intra-articular injection of UC-MSCs improved function and reduced pain in patients with knee osteoarthritis. However, the number of included studies was small and more studies are needed to confirm this.

## 1. Introduction

Knee osteoarthritis (KOA) is a chronic, progressive degenerative disease of the cartilage characterized by progressive cartilage loss, subchondral bone remodeling, bone redundancy formation, and synovial inflammation.^[1]^ Patients suffer from progressive destruction of articular cartilage and surrounding tissues, which are mainly characterized by pain, stiffness, and degenerative joint lesions.^[2]^ The main causative factors are genetic, age, obesity, and nutritional factors. One study showed that the prevalence and incidence of KOA in China were 4.6% and 2.52%, respectively, from 2008 to 2017, and the prevalence increased with age, surging after 55 years of age, with an average incidence of 13.2% in people over 55 years of age.^[3]^ Additionally, KOA increases the risk of other diseases. There was a significant causal relationship between coronary heart disease and KOA.^[4]^ KOA can act as a risk factor for Alzheimer disease, and the systemic inflammation triggered can play a role in the pathogenesis of Alzheimer disease.^[5]^ The pathogenesis of KOA can be understood as a predisposition of the body to excessive mechanical stress that exceeds the carrying capacity of the joint tissues, leading to an imbalance between destruction and repair of the joint tissues, which in turn leads to KOA.^[6]^ Cartilage tissue degeneration is the most typical pathological feature of KOA, and is closely related to chondrocyte apoptosis and extracellular matrix degradation. Under normal conditions, chondrocytes regulate the synthesis and degradation of extracellular matrix components to maintain their balance; however, under pathological conditions, the in vivo balance cannot be maintained, the degradation of the extracellular matrix increases, and the articular cartilage is destroyed.^[7]^ The main function of chondrocytes is to maintain cartilage homeostasis, partly through the production of extracellular matrix components. However, with age, chondrocytes gradually senesce and lose the ability to maintain and repair articular cartilage.^[8]^ It is evident that KOA has a complex etiology with many influencing factors and limited self-repair function. With the prolongation of life expectancy and the increase in obesity, its incidence is bound to increase further. Therefore, timely treatment of KOA is necessary.

Currently, KOA is mostly treated with surgical treatments such as total knee arthroplasty or unicompartmental knee arthroplasty, which are effective treatments for severe KOA at any age, however, many patients are reluctant to undergo surgical treatment.^[9]^ Pharmacological therapy, on the other hand, includes analgesics, nonsteroidal anti-inflammatory drugs, platelet-rich plasma (PRP), hyaluronic acid (HA), and intra-articular corticosteroid injections. In the early stages of the disease, oral analgesics may be used, but only in mild cases, and there is a risk of gastrointestinal disorders. Intra-articular injection of glucocorticoids is an effective treatment for KOA, which can reduce pain in the short term, but there is no reliable basis for long-term pain relief; therefore, long-term use of glucocorticoids is not recommended for patients with KOA.^[10]^ Sodium hyaluronate, whose chemical nature is a macromolecular acidic mucopolysaccharide, is one of the main components of synovial fluid and cartilage matrix, lubricating the joints and protecting the articular cartilage. However, some studies have shown that intra-articular injections of HA provide no difference in pain relief or function from placebo.^[11]^ PRP is a concentrate of highly concentrated platelets obtained from autologous blood and contain high concentrations of growth factors that are known to promote chondrocyte proliferation. A study comparing PRP, HA, and placebo injections for the treatment of KOA found that PRP was more effective than HA and placebo in relieving pain and improving function at short-term follow-up and did not differ from HA or placebo in terms of the risk of adverse events.^[12]^ However, a meta-analysis showed that intra-articular injection of PRP also provided short-term symptomatic relief in patients with KOA; however, its long-term efficacy remains unclear.^[13]^ Stem cells are an effective method to regenerate chondrocytes, which can promote chondrocyte proliferation by balancing the synthesis and degradation of the cartilage extracellular matrix and inhibiting local inflammatory responses in joints.^[14]^ With the continuous increase in mesenchymal stem cells (MSCs) research, MSCs have been found to have unique advantages in repairing cartilage damage, and joint cavity injection of MSCs is expected to become an important therapy for the treatment of KOA. MSCs are adult stem cells that have the ability to differentiate into chondrocytes and osteocytes and play a partial role in the support and maintenance of human connective tissues. MSCs are widely found in human bone marrow, adipose tissue, synovial membranes and other tissues, including bone marrow MSCs (BM-MSCs), adipose MSCs (AD-MSCs), synovial membrane MSCs, and umbilical cord MSCs (UC-MSCs). MSCs are known to have regenerative, anti-inflammatory, immunoregulatory, anti-oxidative stress, anti-fibrotic, antimicrobial, and antitumor effects. With the development and research of MSC exosomes, the exploration of exosomes for use in the treatment of more diseases has begun, and attempts have gradually begun to use exosomes in the treatment of heart disease, kidney disease, orthopedic disease, inflammation and other related diseases.^[15]^ MSCs can directly differentiate into form relevant tissues to supplement the defects of knee cartilage injury sites. On the other hand, MSCs can secrete cytokines to promote the regeneration of cells around them when they are subjected to the action of the surrounding microenvironment, which is known as the trophic effect, and this paracrine effect on the neighboring cells is the main pathway to promote the repair of cartilage.^[16–18]^ In addition, MSCs may also be involved in the regulation of T-cell-related immune responses, which further suppresses osteoarthritis-related inflammatory responses, thereby slowing the progression of KOA.^[19]^ A study evaluated the latest clinical data and published research articles between 2014 and 2019 and found that BM-MSCs have multilineage differentiation potential, immunosuppressive ability, and can be used for clinical treatment of KOA.^[20]^ At present, BM-MSCs and AD-MSCs are widely used in the treatment of KOA by intra-articular injection, but there are certain limitations, such as the difficulty of obtaining BM-MSCs and the greater harm to the donor, which limits their clinical application, and the AD-MSCs, they are easier to obtain, simple to isolate, and with higher yields, have disadvantages such as poor osteogenic and chondrogenic potential.^[21]^ In recent years, owing to the advantages of convenient umbilical cord collection, lack of harm to mothers and newborns, less ethical controversy, high differentiation potential, high in vitro proliferation capacity, and batch preparation for quality control, more and more attention have received increasing to UC-MSCs. A study suggested that UC-MSC-exosomes can suppress inflammation and prevent degeneration of articular cartilage and synovial tissue by inhibiting pro-inflammatory cytokines.^[22]^ UC-MSCs induced the secretion of some cytokines and activated the Wnt/β-catenin signaling pathway through paracrine function to alleviate the pathological condition of KOA and maintain the normal expression of cytokines and extracellular matrix proteins.^[23]^ In recent years, clinical studies on the use of UC-MSCs for the treatment of KOA have been reported. After 7 years of follow-up, one study concluded that the injection of umbilical cord MSCs can promote the regeneration of hyaline-like cartilage in KOA and is safe and effective.^[24]^ Clinical trials have been conducted to evaluate the efficacy of intra-articular injections of UC-MSCs. Dilogo summarized the clinical efficacy and safety of UC-MSCs, but included animal studies and the evidence was not sufficiently rigorous.^[25]^ Therefore, the present study evaluated the efficacy of intra-articular injection of UC-MSCs in KOA by including a clinical randomized controlled trial investigator with the aim of obtaining high quality clinical evidence.

## 2. Methods

This meta-analysis followed the Preferred Reporting Items for Systematic Reviews and Meta-Analyses guidelines^[26]^ and was registered on PROSPERO (CRD42024545484). Given that this study was not a clinical trial, ethical approval was not required.

### 2.1. Eligibility criteria

This study included a randomized controlled pilot study of KOA with intra-articular injection of UC-MSCs.

#### 2.1.1. Inclusion criteria

- Studies that included only patients with diagnosis KOA.
- The study compared intra-articular injections of UC-MSCs with other conventional methods for the treatment of KOA.
- The study included at least one of the following assessment instruments: Western Ontario and McMaster Universities Osteoarthritis Index (WOMAC) or Knee Lysholm Score (KLS).

#### 2.1.2. Exclusion criteria

- Animal studies, reviews, and meta-analyses.
- Not a randomized controlled trial.
- No randomized controlled trials have compared intra-articular injections of UC-MSCs with other treatments for KOA.
- Articles in which WOMAC or KLS was not used as an outcome measure.
- Studies in which raw data were lacking or data could not be extracted and transformed.
- There isn’t an appropriate number of patients to justify the study.

### 2.2. Literature search strategy

PubMed, Web of Science, Cochrane Library, Embase, Chinese National Knowledge Infrastructure, and Wanfang databases were searched from database creation to March 31, 2024. A combination of MeSH terms and free words was used for the search, and the search strategy was based on the characteristics of each database.

### 2.3. Data extraction and biased risk assessment

Two authors extracted data from the selected studies, including author names, year of publication, sample size, number of participants in the experimental and control groups, mean age of participants, country, diagnosis, intervention and control, cell dose, duration of treatment, and outcome measures.

Two authors in the included studies assessed the risk of bias using the Physiotherapy Evidence Database (PEDro) scale.^[27]^ Discrepancies were resolved through discussion or arbitration by a third evaluator. The total PEDro score was obtained by summing the scores for items 2 through 11, with the total score ranging from 0 to 10. Higher scores indicated higher methodological quality. A score of <4 was considered “poor,” 4 to 5 as “fair,” 6 to 8 as “good,” and 9 to 10 as “excellent.”

### 2.4. Outcome measures

The primary outcome measure in this meta-analysis was the WOMAC score. The secondary outcome measure was the KLS.

### 2.5. Data analysis

Data were analyzed using RevMan software (version 5.3). The 95% confidence intervals (CI) of the combined mean difference (MD) were used to assess differences in outcomes between patients with KOA who received UC-MSCs and those who received other forms of treatment. The chi-squared test was used to analyze and quantify the magnitude of heterogeneity in the included studies. *I*^2^ > 50% indicates that heterogeneity exists and the source of heterogeneity needs to be analyzed, and *I*^2^ < 50%, indicates that no heterogeneity exists, indicating a good concordance of results. Additionally, it is necessary to choose an appropriate method for combining effect sizes according to the magnitude of heterogeneity. If the heterogeneity was large (*I*^2^ > 50%), the meta-analysis was performed using a random effects model, otherwise, the analysis was performed using a fixed effects model (*I*^2^ < 50%). Statistical significance was set at *P* < .05.

## 3. Results

### 3.1. Search result

**Figure 1.**
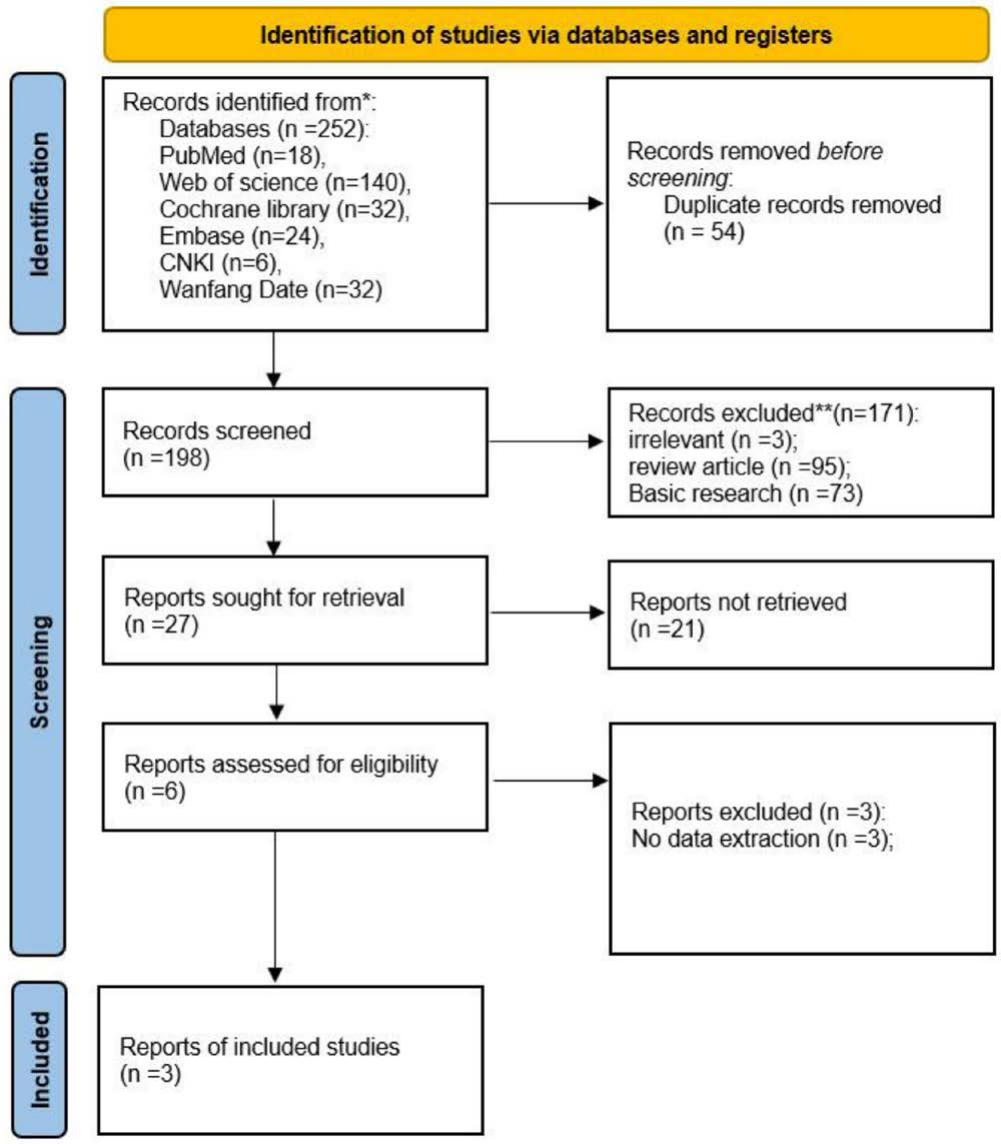
Flow diagram of study selection and identification.

We searched 6 databases, PubMed, Web of Science, Cochrane Library, Embase, Chinese National Knowledge Infrastructure, and Wanfang, and included 293 studies related to the treatment of KOA with UC-MSCs. Three studies were included according to the inclusion and exclusion criteria, 3 studies were finally included^[28–30]^ (Fig. 1).

### 3.2. Study characteristics

The 3 studies involved 101 participants, with sample sizes ranging from 17 to 48. All studies used a single treatment (UC-MSCs) with cell injections ranging from 20 × 10^6^ to 30 × 10^6^. Two studies used HA treatment in the control group, and one study used NS treatment. The treatment duration was lasted at least 6 months, and up to 12 months. Three studies used WOMAC as the primary outcome indicator and 2 studies used KLS as the outcome indicator (Table 1).

**Table 1:** The basic characteristics of included studies.

References	Sample size	T/C (M/F)	Age (years)	Country	Diagnosis standard	Intervention	Control	Cell dose	Treatment duration	Outcome measures
Matas J et al 2019^[28]^	17	T:9 (3/6) C:8 (3/5)	T:56.7 ± 4.1 C:54.8 ± 4.5	Chile	KOA	UC-MSCs	HA	20 × 10^6^	12 months	a
Sun Y et al 2022^[29]^	48	T:24 (20/4) C:24 (15/9)	T:35 ± 5 C:35 ± 5	China	KOA	UC-MSCs	NS	20 × 10^6^	12 months	a, b
Wang YL et al 2016^[30]^	36	T:18 (10/8) C:18 (11/7)	T:54.28 C:52.37	China	KOA	UC-MSCs	HA	20–30 × 10^6^	6 months	a, b

a: WOMAC; b: KLS; c: control.

F = female, HA = hyaluronic acid, KLS = Knee Lysholm score, KOA = knee osteoarthritis, M = man, NS = normal saline, T = treatment, UC-MSCs = umbilical cord mesenchymal stem cells, WOMAC = Western Ontario and McMaster Universities Osteoarthritis Index.

### 3.3. Risk of bias assessment

Two authors (ZJX and XYW) independently assessed the quality of included studies using the PEDro tool. The mean PEDro score in the included studies was 7.67. All studies had a score greater than or equal to 6, indicating a low risk of bias (Table 2).

**Table 2:** Physiotherapy evidence database (PEDro) scores of the 3 included studies.

References	Eligibility criteria	Random allocation	Concealed allocation	Baseline comparability	Blind subjects	Blind therapists	Blind assessor	Adequate follow-up dropout < 15%	Intention-to-treat analysis	Between-group comparisons	Point estimates and variability	Score
Matas J et al 2019^[28]^	1	1	1	1	1	1	1	1	1	1	1	10
Sun Y et al 2022^[29]^	1	1	1	1	0	0	0	1	1	1	1	7
Wang YL et al 2016^[30]^	1	1	0	1	0	0	0	1	1	1	1	6
Mean												7.67

### 3.4. Meta-analysis results

#### 3.4.1. Western Ontario and McMaster Universities Osteoarthritis Index

**Figure 2.**
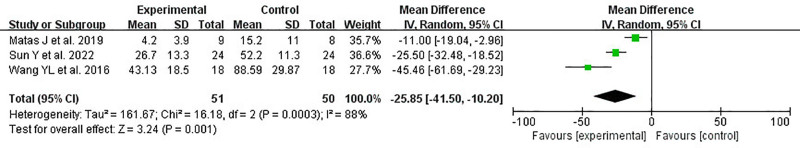
Forest plot of WOMAC. WOMAC = Western Ontario and McMaster Universities Osteoarthritis Index.

A meta-analysis showed that intra-articular injections of UC-MSCs can significantly improve function in patients with KOA. WOMAC was described in 3 studies, which included 101 patients (51 in the UC-MSCs group and 50 in the control group). WOMAC was reduced in the UC-MSCs group compared to the that in control group (MD: ‐25.85; 95% CI: −41.50, −10.20; *P* = .001). In addition, the results showed large heterogeneity (*I*^2^ = 88%) (Fig. 2).

#### 3.4.2. Knee Lysholm Score

**Figure 3.**
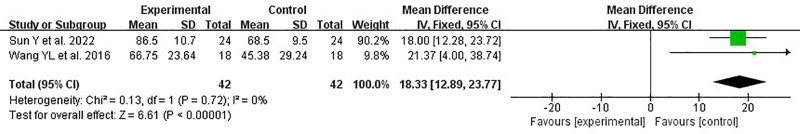
Forest plot of KLS. KLS = Knee Lysholm Score.

The results of the meta-analysis showed that intra-articular injection of UC-MSCs was significantly associated with for pain in patients with KOA. KLS was described in 2 studies that included 84 patients (42 UC-MSCs and 42 control groups). KLS was improved in the UC-MSCs group compared with the control group (MD: 18.33; 95% CI: 12.89, 23.77; *P* < .00001). In addition, the results showed no heterogeneity (*I*^2^ = 0%) (Fig. 3).

#### 3.4.3. Publication bias

**Figure 4.**
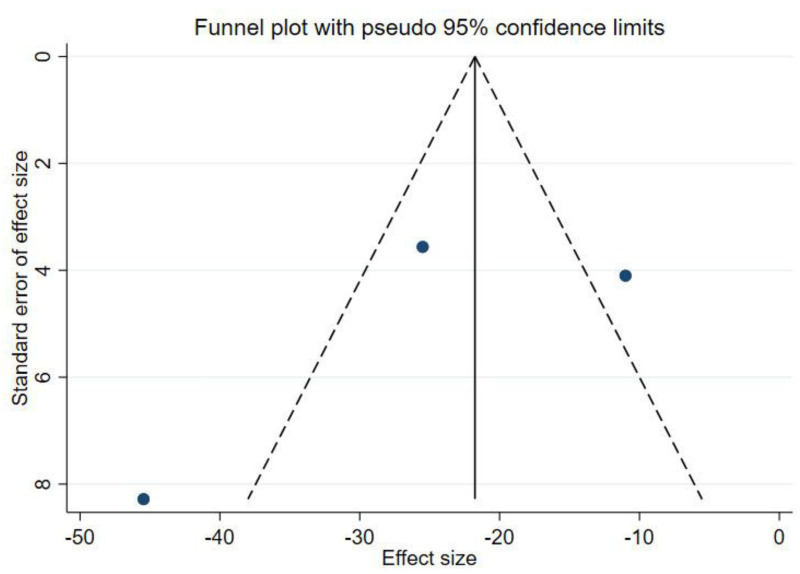
Funnel plot of WOMAC. WOMAC = Western Ontario and McMaster Universities Osteoarthritis Index.

**Figure 5.**
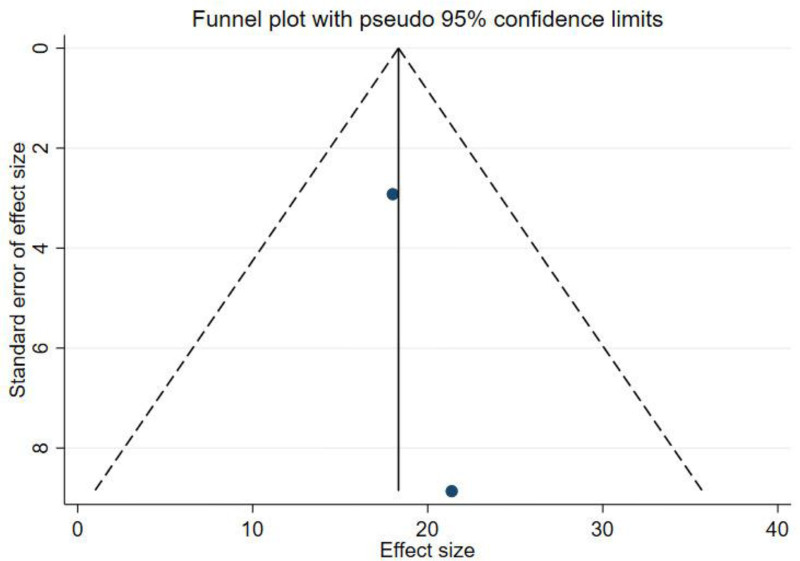
Funnel plot of KLS. KLS = Knee Lysholm Score.

**Figure 6.**
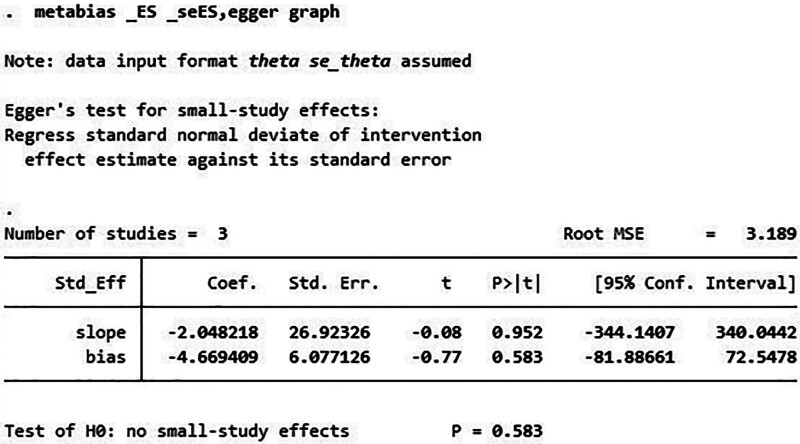
Egger test of WOMAC. WOMAC = Western Ontario and McMaster Universities Osteoarthritis Index.

We performed a publication bias analysis of the included studies, and the funnel plot showed that the WOMAC was asymmetric, whereas the KLS was symmetric, suggesting that there may be a publication bias in the WOMAC results (Fig. 4, Fig. 5). Therefore, we performed the Egger test. The results showed *P* = .583, indicating that there was no publication bias (Fig. 6).

#### 3.4.4. Sensitivity analysis

**Figure 7.**
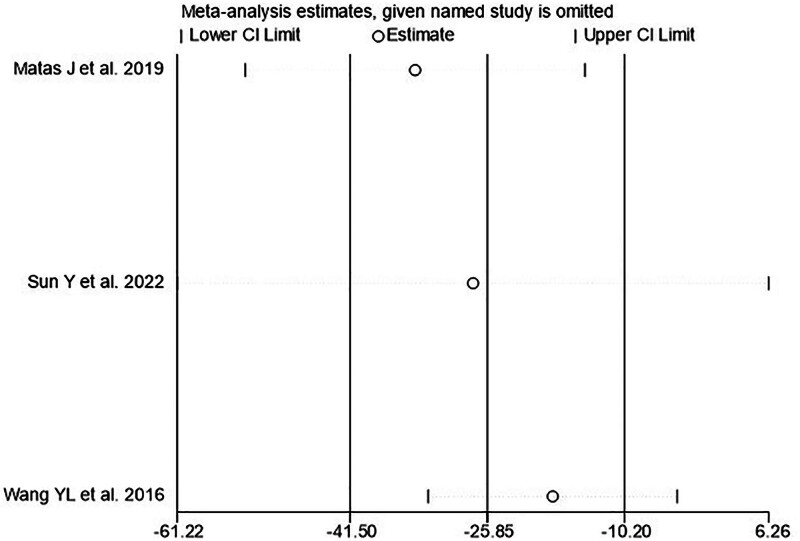
Sensitivity analysis of WOMAC. WOMAC = Western Ontario and McMaster Universities Osteoarthritis Index.

The results of the WOMAC meta-analysis showed was high heterogeneity, and subgroup analysis could not be performed due to the small number of included studies; therefore, we performed a sensitivity analysis. The results showed that after excluding the literature individually, the result was still within the 95% CI, indicating that the result was stable (Fig. 7).

## 4. Discussion

KOA is a degenerative disease of the joints. For insignificant degeneration, mild symptoms, and small cartilage damage, nonsurgical treatment is usually used clinically. However, for obvious degeneration, severe symptoms, and serious cartilage damage, surgical treatment is usually used. The initial intention of conservative treatment is mainly to delay the timing of surgery based on symptomatic relief. MSCs are stem cells with self-replicating and multidirectional differentiation potential, and are widely distributed in human tissues. MSCs can able to be induced to differentiate into different tissue cells, such as chondrocytes, synoviocytes, and adipocytes, in specific environments.^[31]^ Therefore, MSCs from various sources have been widely used to repair diseases in terms of tissue and organ damage caused by aging and lesions, such as bone marrow injury or trauma repair. Orozco used BM-MSCs for intra-articular injections, and at 12 months, the patients’ pain and quality of life significantly improved.^[32]^ Pak used AD-MSCs for the treatment of KOA and found significant improvements in pain and mobility.^[33]^ MSCs have a promising future in regenerative medicine; however, BM-MSCs, although widely used, are significantly hindered in application owing to difficulties in obtaining them and greater donor injury. AD-MSCs, although abundantly sourced, have poor in terms of their osteogenic and chondrogenic potentials. UC-MSCs have high potential for differentiation, high proliferative capacity in vitro, low immunogenicity, and are less harmful to mothers and neonates, which makes them able to replace BM-MSCs and AD-MSCs.^[34]^ Mennan concluded that UC-MSCs had the best differentiation capacity, and compared to BM-MSCs, UC-MSCs induced the production of fewer adipocytes, suggesting that, under the same conditions, UC-MSCs induced differentiation to produce more osteoblasts or chondrocytes.^[35]^ Tong showed that UC-MSCs treatment can maintain the function of cartilage surface cells and inhibit the inflammatory response, thus slowing the progression of KOA.^[36]^ Lim conducted a 48-week clinical trial on 114 patients with total cartilage defects of the knee joint, implanted with UC-MSCs and HA by mini-arthrotomy, and the visual analog score and WOMAC were found to be significantly improved.^[37]^ UC-MSCs are effective in the treatment of KOA, and may play a therapeutic role by promoting chondrocyte regeneration, inhibiting inflammatory responses, and regulating immune tolerance.

Three trials were included in this study to analyze the efficacy of UC-MSCs in the treatment of KOA. Meta-analysis showed that both the WOMAC and KLS of KOA patients treated with UC-MSCs improved significantly compared with those of the control group, suggesting that UC-MSCs can improve the function and pain of KOA. Egger test suggested no publication bias. However, there was heterogeneity in the WOMAC results, and the results were suggested to be stable by the sensitivity analysis. Therefore, the high heterogeneity may be related to the small number of included studies or the insufficient sample size. Adverse events, including pain and joint effusion after injection, were mentioned in 2 studies; however, they were all transient, and no serious adverse events occurred.

This study had some limitations. First, only 3 randomized controlled trials were included in this study, all of which were single-center, small-sample clinical studies, and lacked multicenter, large-sample trials. Second, the clinical trials collectively used fewer outcome indicators to evaluate the treatment effects of UC-MSCs from more perspectives, and both WOMAC and KLS were subjective assessment indicators that may have measurement bias.

## 5. Conclusion

By including randomized controlled trials for meta-analysis, our results suggest that intra-articular injection of UC-MSCs improved pain and function in patients with KOA, but its long-term efficacy is still unclear and needs further evaluation.

## Author contributions

**Conceptualization:** Zhijian Xiao, Lihua Luo.

**Data curation:** Cheng Li, Wei Li.

**Formal analysis:** Cheng Li.

**Funding acquisition:** Zhijian Xiao, Lihua Luo.

**Investigation:** Cheng Li, Wei Li.

**Methodology:** Zhijian Xiao, Xinying Wang.

**Project administration:** Zhijian Xiao, Lihua Luo.

**Software:** Zhijian Xiao, Xinying Wang, Wei Li.

**Supervision:** Zhijian Xiao, Lihua Luo.

**Validation:** Zhijian Xiao, Xinying Wang, Cheng Li, Lihua Luo.

**Visualization:** Zhijian Xiao.

**Writing – original draft:** Zhijian Xiao, Xinying Wang.

**Writing – review & editing:** Zhijian Xiao, Xinying Wang.
